# Screening for cataract in the community

**Published:** 2026-07-22

**Authors:** Andrew Blaikie, Lila Raj Puri

**Affiliations:** 1 Consultant Ophthalmologist: NHS Fife, St Andrews, Scotland; Senior Lecturer: University of St Andrews, St Andrews, Scotland; Honorary Associate Professor: International Centre for Eye Health, London School of Hygiene & Tropical Medicine, UK.; 2 Medical Advisor, Asia: The Fred Hollows Foundation, London, UK.

**Age-related cataract is the most common cause of treatable blindness globally, so it is vital that patients with visually significant cataract are identified and referred promptly.**

**Figure:**
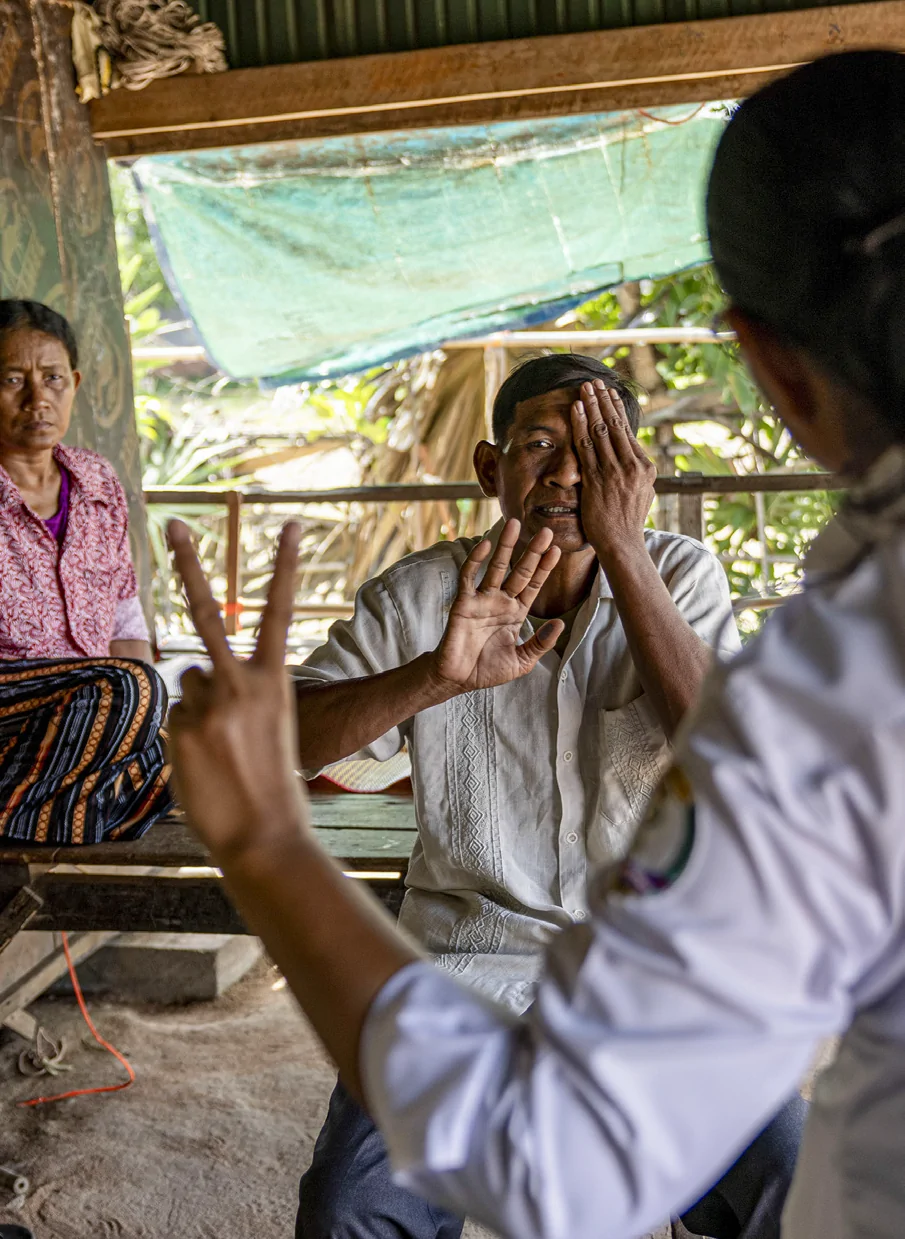
Screening patients for cataract in the community. CAMBODIA

© MICHAEL AMENDOLIA/THE FRED HOLLOWS FOUNDATION CC BY-NC-SA 4.0

Screening for cataract in the community is vital in order to ensure that under-served and high-risk populations are able to undergo safe, high-quality surgery. Community screening should prioritise:

- Adults aged 50 years and older
- Those reporting difficulty in carrying out daily activities
- Women, people on low incomes, people living with disabilities, and people living in remote areas.

Due to the costs, time, and often long distances involved in having cataract surgery, it is important to avoid referring patients who do not have treatable cataract.

In this article, we suggest how, even with minimal equipment and instruments, eye health workers at community level can refer those patients most likely to benefit from surgery, while also keeping a careful eye out for ocular emergencies or other eye conditions that require referral and treatment.

Briefly, this can be done by:

1. Confirming there is significant sight loss, i.e., visual acuity (VA) < 6/12 or 6/18, depending on local guidelines.
2. Checking that the patient’s history and the results of other examinations are consistent with cataract.
3. Ruling out other causes of sight loss, and referring these patients to specialist services where needed.

## What you will need

- A near and distance visual acuity chart
- Measuring tape or string of known length to position the chart and patient at the correct distance apart
- An occluder with a pinhole
- A pen torch or Arclight device
- Referral slips, data sheets, and a pen.

To effectively screen for cataract, you will ideally need access to a room where you can **make the room as dark as possible**, e.g., by covering the windows. This is so you can check pupil response and test the fundal reflex (glow) effectively.

However, there is still a lot you can do and find out, even if you cannot make the room very dark.

## Initial examination

### 1. Take a history

Ask about the patient’s age, lifestyle, and their medical history:

- How old are you?
- Do you smoke, or spend a lot of time outdoors?
- Do you have any past history of eye problems or treatment (e.g., eye injury, use of spectacles, medication/surgery or eye problems as a child)?

Sight loss due to age-related cataract usually affects people aged 50 years or older; however, lifestyle factors such as smoking and high levels of UV exposure can lead to earlier onset.^1^ It is also possible to develop cataract due to an eye injury; this is usually in one eye only and the onset can be quicker, and there can be additional signs on examination, such as a corneal scar or a misshapen pupil.

Ask about the sight loss:

- Has it been gradual or sudden? Sight loss due to age-related cataract is painless and gradual. Urgently refer patients with sudden sight loss or pain to an eye specialist.
- Are one or both eyes affected? Age-related cataract usually affects both eyes, but it is often worse in one eye.
- Has there been any pain and/or redness? Patients with vision loss accompanied by pain and redness need to be seen by an eye specialist promptly, as they may have a more urgent sight-threatening condition. They may still have cataract, but this would be detected by the eye specialist.
- Is sight loss interfering with your daily activities? People who have difficulty with their daily living activities because of cataract are most likely to benefit from surgery.

Ask about other symptoms:

- Do you experience discomfort when looking at bright lights, particularly at night? Glare can be one of the early signs of cataract.

### 2. Assess visual acuity

- Test and record distance visual acuity in each eye separately; first unaided, then with the pinhole occluder.
- Test and record near visual acuity. If near vision is reduced, tell the person they would benefit from near-vision spectacles, and explain where to get them.

**What this means.** People who are likely to benefit from cataract surgery will have reduced vision. The World Health Organization now measures the effectiveness of a country’s cataract surgery by the number of people able to see better than 6/12 after their operation. By implication, people with visual acuity of less than 6/12 should be offered surgery. However, in many countries, surgery is only offered to people with visual acuity of less than 6/18; this may also depend on the hospital or context where surgery is done.

If vision improves with pinhole, or if they have reduced near vision, they may need distance vision and/or near-vision spectacles and should be referred for refraction. If they also have cataract, their refractive error will change after surgery, depending on the intraocular lens used. These patients should be referred for refraction following surgery; this is usually done at or after the last follow-up visit.

### 3. Examine the front of the eye

Examine the front of the eye using a torch (ideally with some form of magnification) or an Arclight. The following patients should be referred urgently to an eye specialist:

- Patients with any sudden vision loss or history of eye trauma (e.g., Figure 1A)
- Patients with a painful red eye, with discharge (e.g., Figure 1B) or without discharge (e.g., Figure 1C)
- A young patient with a white pupil

**Figure 1:**
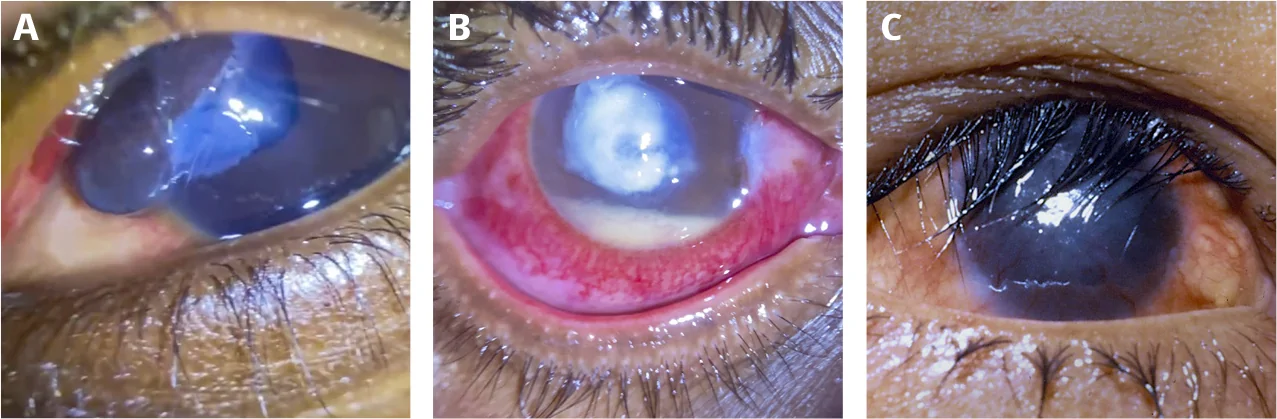
Examples of patients who need urgent referral: **A.** Trauma with iris prolapse. **B.** Infective keratitis. **C.** Trachoma with trichiasis.

© ANDREW BLAIKIE, UNIVERSITY OF ABERDEEN CC BY-NC-SA 4.0

A corneal scar can be confused with a cataract.

- **Cataract** can appear as a central, round, grey/white area filling the whole of the pupil space (Figure 2A).
- **Corneal scars** are on the surface of the eye (the cornea) and are often more irregular in shape; they are not always central (Figure 2B).

**Figure 2:**
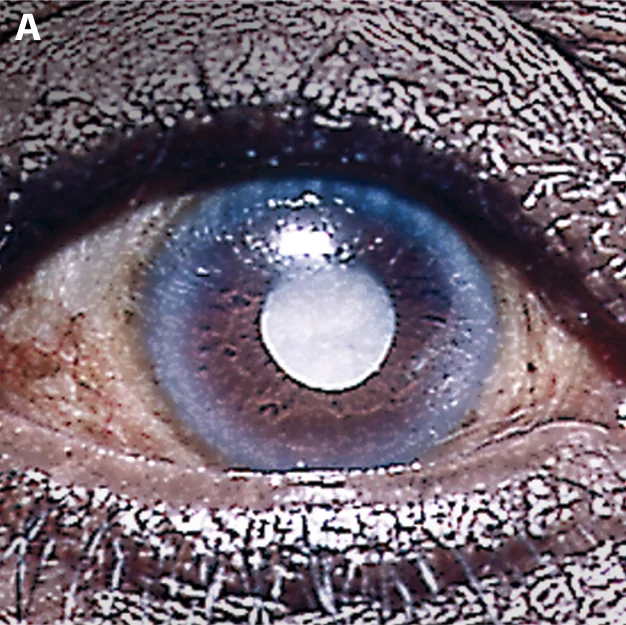

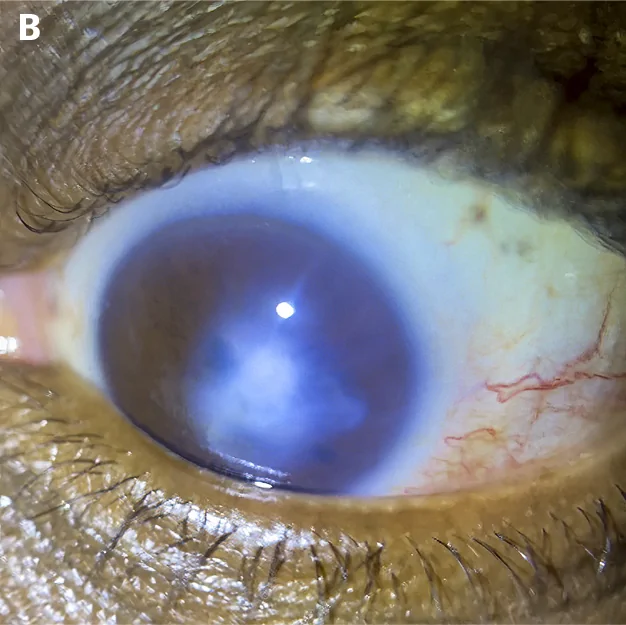
Comparison of eyes with cataract (**A**) and corneal scar (**B**).

© ANDREW BLAIKIE, UNIVERSITY OF ABERDEEN CC BY-NC-SA 4.0

© ANDREW BLAIKIE, UNIVERSITY OF ABERDEEN CC BY-NC-SA 4.0

If the corneal scar is central, and/or significantly obscures vision, the patient would benefit from being referred to an eye specialist.

### 4. Look at the pupil

- Are the pupils round and equal? Are both pupils the same size? If not, refer the person to a medical doctor or eye care professional to rule out a more urgent condition.
- Are the pupils black? In some patients with mature cataract, the pupil can look grey or white (Figure 3). However, operable cataract is not always visible as a grey or white pupil. Posterior subcapsular cataract is a good example: the anterior examination may look fairly normal, but the patient can have poor vision and experience significant glare when looking at bright light or driving at night.

**Figure 3:**
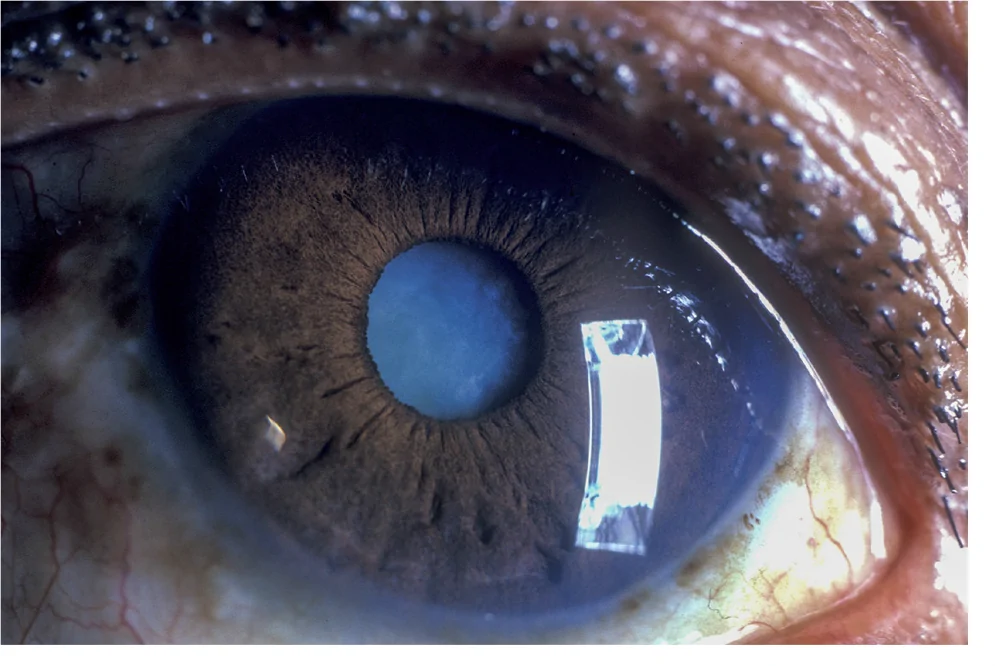
In patients with cataract, the pupil can look grey or white.

© ANDREW BLAIKIE, UNIVERSITY OF ABERDEEN CC BY-NC-SA 4.0

## If you can make the room dark

### 1. Check the pupil response to bright light

Do the following:

- Ensure the room is as dim as possible (e.g., turn off the light and cover any windows).
- Position the patient so that any remaining source of light is behind them, and they are looking into the darkest part of the room.
- Turn on your torch or Arclight. Shine it at one eye at a time. Each pupil should constrict briskly, even if cataract is present.

**What this means.** In patients with cataract, even significant cataract, the pupil should constrict normally. If it does not respond or constrict, this suggests an optic nerve problem such as advanced glaucoma, optic atrophy, or retinal disease (such as retinal detachment). Refer these patients to an eye specialist. They may still benefit from cataract surgery, but the problem at the back of the eye needs to be assessed first.

### 2. Check the fundal reflex

If a torch or Arclight is available, the fundal reflex test can add useful information and improve the quality of the referral.

What to do:

- Keep the lights off and windows covered so the room continues to be dark
- Stand an arm’s length away from the patient and hold the torch or Arclight at their eye level, pointing at the midpoint between the eyes.
- Ask the patient to look at the light or just above it, so the light is shining into both pupils at the same time. The fundal reflex is the light that reflects from the back of the eye, visible through the pupil (Figure 4).
- Observe and compare the reflex in each eye, with reference to the VA recorder earlier. Note whether the reflex is the same in both eyes, or whether it is dull or completely absent in one or both eyes. If there is a difference between the eyes, write down which eye has the brighter reflex.

**Figure 4:**
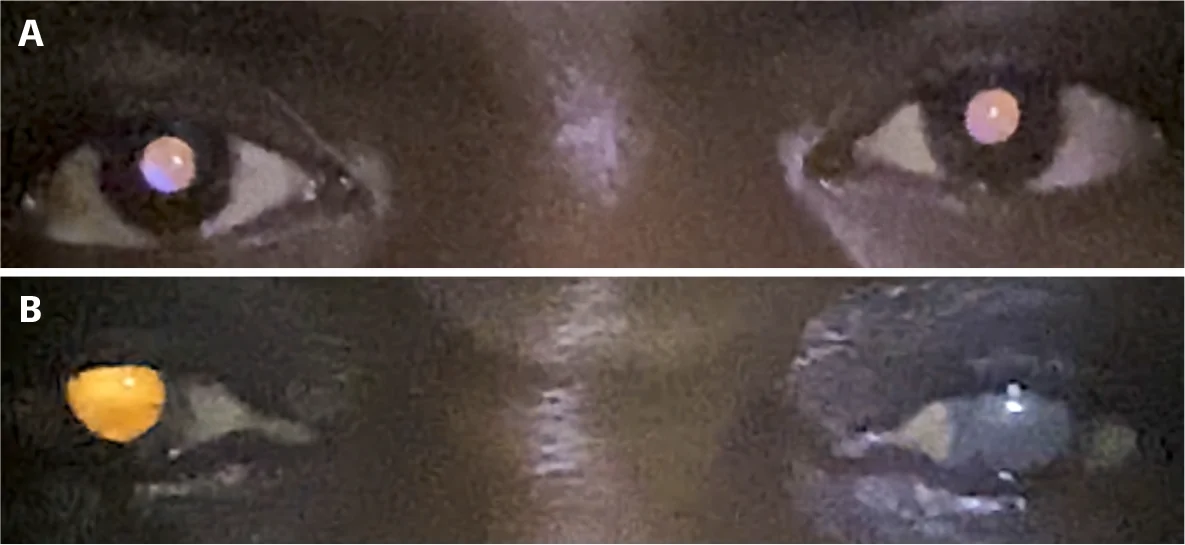
Normal and abnormal fundal reflex. **A.** Normal fundal reflex in both eyes. **B.** Normal reflex in the right eye, with absent reflex in the left eye due to unilateral cataract (this person’s eyes were dilated before examination).

© ANDREW BLAIKIE, UNIVERSITY OF ABERDEEN CC BY-NC-SA 4.0

**What this means.** In patients with cataract, the fundal reflex should be abnormal or absent, and the brightness of the reflex should correspond inversely to the degree of opacity of the cataract.

- In the absence of cataract, the brightness and colour will be the same in both eyes (Figure 4A).
- If the patient has unilateral cataract (often due to an eye injury), the reflex may be dull or even completely absent in the affected eye (Figure 4B).
- The reflex can be abnormal in other conditions too, such as a retinal detachment or a bleed from diabetic retinopathy; consider the patient’s history.
- In patients with age-related cataract, the fundal reflex should appear brighter in the eye with better VA; this is because the cataract in that eye is less dense, allowing more light to pass through. Therefore, if the visual acuity and fundal reflex results are consistent with each other (i.e., the eye with the brighter reflex also has higher visual acuity), this means cataract is the most likely cause.

### Counselling and referral

Explain the benefits and risks of cataract surgery, where to go, and what is involved, including the cost. This will include travel, fee for surgery, food and water, and the indirect costs of time away from work or home. The person is likely to need someone to accompany them and help with postoperative care too.

Complete any clinical documentation and give the patient a referral slip with the address of the clinic and the date and time they must attend. Record the patient’s phone number and other relevant details so you can follow up and track those who do not attend.

**Should I refer a patient for cataract surgery?**

In general, it is advisable to refer patients for cataract surgery if:

- They have reduced vision (<6/18 or <6/12), consistent with age-related cataract, that causes difficulties with daily functioning.
- There is a visible lens opacity or an abnormal fundal reflex.
- There is no obvious alternative explanation (i.e., nothing is visible on torch light examination, and the pupil response is normal).

However, **if you cannot make the room dark**, refer patients whose history is consistent with cataract, who have no other apparent causes of sight impairment, and who have visual acuity with pinhole worse than 6/12 to 6/18 (depending on the referral criteria for your area).

**If you can check the pupil response to bright light**, refer patients who meet the above criteria and have a normal pupil response; those with an abnormal response should be referred to an eye specialist before cataract surgery is considered.

**If you can also check the fundal reflex**, refer patients who also have an abnormal fundal reflex that is consistent with the visual acuity results.

All referred patients still need a more detailed examination before surgery – see the article in issue 128: ‘Assessing patients at the cataract treatment centre and allocating the right surgeon’ (bit.ly/cehj885).
